# Recruitment of Refugees for Health Research: A Qualitative Study to Add Refugees’ Perspectives

**DOI:** 10.3390/ijerph14020125

**Published:** 2017-01-29

**Authors:** Patricia Gabriel, Janusz Kaczorowski, Nicole Berry

**Affiliations:** 1 Department of Family Practice, University of British Columbia, Vancouver, BC V6T 1Z3, Canada; 2 Department of Family and Emergency Medicine, University of Montreal, Montreal, QC H3T 1J4, Canada; januszk@gmail.com; 3 Faculty of Health Sciences, Simon Fraser University, Burnaby, BC V5A 1S6, Canada; nsb4@sfu.ca

**Keywords:** refugee, recruitment, patient, qualitative research, ethics, research

## Abstract

Research is needed to understand refugees’ health challenges and barriers to accessing health services during settlement. However, there are practical and ethical challenges for engaging refugees as participants. Despite this, there have been no studies to date specifically investigating refugee perspectives on factors affecting engagement in health research. Language-concordant focus groups in British Columbia, Canada, with four government-assisted refugee language groups (Farsi/Dari, Somali, Karen, Arabic) inquired about willingness to participate in health research. Twenty-three variables associated with the willingness of refugees to participate in health research were elicited. Variables related to research design included recruitment strategies, characteristics of the research team members and the nature of the research. Variables related to individual participants included demographic features such as gender and education, attitudes towards research and previous experience with research. This research can be used to increase opportunities for refugees’ engagement in research and includes recommendations for subgroups of refugees that may have more difficulties engaging in research.

## 1. Introduction

Refugees have complex migration histories leading to health concerns prior to, during, and after settlement in their new host countries. Timely access to appropriate health care is essential to both physical and mental wellness and the ability to settle successfully. Unfortunately, there are barriers to accessing health care for refugees at the health system, provider and individual level. These barriers often translate into decreased utilization of needed health care services [1] and ultimately refugees can experience a decline in health status after arrival, even in resource-rich nations such as Canada [2]. To address this disparity, research is needed to understand refugees’ health challenges and barriers to accessing health services.

However, to do such research, one first must understand that engaging refugees in research studies can be challenging due to a number of barriers [3]. For many refugee populations there may be obvious challenges due to communication difficulties as a result of language and cultural barriers or low literacy levels [4]. Potential participants can be difficult to recruit and follow due to refugees being a harder to reach and highly-mobile population [5]. These necessitate a need to adapt recruitment and study materials, and consider alternative means of contacting potential participants. There are also less tangible challenges, such as how to address potential higher rates of distrust, fear, and suspicion [6,7], being “socially invisible” [8], potential power imbalances, institutional discrimination, and trauma associated with pre-migration, migration, and settlement experiences [9] that create ethical challenges to obtaining true informed consent.

To date, understanding of these research challenges has focused primarily on obstacles perceived by researchers and identified as part of studies designed for other purposes [2,10,11]. Academics in the field have made thoughtful suggestions for improving the efficacy and effectiveness of research with refugees [6,8,9,12] while ensuring the ethical appropriateness of the methods [13].

However, only a few studies have directly sought the opinions of ethnic minority or refugee participants on the research process. With respect to ethnic minority groups broadly, there were three studies found inquiring with participants about barriers and enablers to participation in research. A 2012 German study [14] with Turkish migrants, a 2005 Canadian study [15] with migrant women, and a 2003 American study [3] of African American, Latino and Chinese Americans all used qualitative methods to elicit participant perspectives on research. These studies, by adding participants’ voices, have resulted in greater understanding of ethnic minority participants’ willingness to engage in health research and suggestions have been made for culturally appropriate research designs for minority participant involvement [2,4,5,8,10,14,16]. With respect to the opinions of refugees in particular, one longitudinal cohort study of refugee youth in Australia [17] asked refugee participants about their experiences having engaged with the research study for many years. The feedback was positive, in particular about opportunities to develop interpersonal relationships, to learn, to have fun, to self-reflect and to acquire small tangible rewards such as cinema tickets. However, this feedback was limited to youth participants and their reflections on having participated in a particular study about settlement and wellness.

Thus, the knowledge base for recommendations about conducting research with refugees still lacks input directly from a broad range of refugee participants and on the topic of factors influencing willingness to participate in health research. Without refugees’ perspectives, our understanding of the factors that impact willingness to participate in research will remain limited in depth and breadth. The current study clearly documents refugee participants’ opinions on factors that may impact refugees’ willingness to participate in health research. This study additionally explored refugees perspectives on ethical challenges in health research which are reported elsewhere [18]. This body of research will serve to improve the effectiveness and ethical appropriateness of refugee research.

## 2. Materials and Methods

Focus group sessions hosted by language-concordant research assistants (RAs) were held in British Columbia (BC) Canada in 2011, with the four most locally prevalent government-assisted refugee (GAR) language groups (and country of origin) at that time being Arabic (Iraq), Farsi/Dari (Afghanistan), Karen (Burma) and Somali (Somalia). Of note, GARs (also known as convention refugees) in Canada are selected abroad through the United Nations UN High Commission for Refugees (UNHCR) and are granted permanent residency status on arrival in addition to health care coverage and a year of financial support. These refugees often come from areas of prolonged conflict and many GARs have spent years in refugee camps with substandard housing, food, education and health care.

The main recruitment sites for this study were two community health care centers that serve refugees near Vancouver BC. Four additional recruitment strategies were used including cold calls to health-clinic patients, invitations to personal contacts of the research assistants, recruitment at refugee-focused community centers and snowball sampling. Inclusion criteria for the focus groups included GARs in Canada for less than five years who were older than 19 and who spoke one of the focus group languages. Participants were offered lunch, childcare, transportation reimbursement of $5, and an honorarium of $20.

Four language-concordant RAs were hired to aid in recruitment and to facilitate focus group sessions. During the focus groups, RAs utilized a semi-structured interview guide to inquire about participants’ attitudes towards, knowledge about, and experience with research and willingness to participate in research. A case study was presented involving a research assistant approaching a newly-arrived refugee to participate in a health-related research study. Participants were then invited to consider alternative iterations of the case study where elements such as characteristics of the refugee, the type of research study, the identity of the research assistant, and the information provided were different from the original case study.

The focus group sessions were audiotaped and transcribed. Results were analyzed using coding and thematic analysis by the lead researcher with input from the four RAs. Ethical approval for this research was obtained from the University of British Columbia Behavioural Research Ethics Board (H10-02296).

## 3. Results

Forty-eight participants attended the four focus groups. A total of 73 participants were invited. The average age was 42 (range from 19 to 65) and 76% were female. About half reported speaking some English, with the rest having no English language skills. Half of participants had no formal education or primary education only. Twenty percent had been in Canada for less than one year. Twenty-three variables were found that impacted refugees’ willingness to participate in research. These are summarized in Table 1 and elaborated on below.

### 3.1. Research Design Factors

#### 3.1.1. Recruitment

Practical aspects of recruitment, such as language, timing and providing financial incentives, in addition to how information about the study is communicated, influenced willingness to participate. If recruitment took place in the first month in Canada, participants felt an individual would be unable to think clearly about participation. They may participate without fully understanding due to fear or a perceived sense of obligation. Alternatively they may decline due to suspicion or fatigue. Most participants indicated that they would be more likely to understand and engage willingly after a longer period of time. Importantly, they felt that after a longer duration of time they would be more likely to understand and engage willingly, rather than out of a perceived sense of obligation. An Arabic male said “As a newcomer, everything would be new, he might be confused. I think he would refuse to participate”. A Somali female added “for me if a researcher came on my first day with an interpreter, I would probably cooperate more. But after a week, chances are people have told me so many things, and they have warned me a lot and planted so much fear in me that I will not cooperate”. A Farsi female commented “bit by bit as time goes on, she talks to one, two or other people and interacts with the environment and starts feeling satisfied with her home. After all this she is then ready to participate in a project in order to learn something”.

Unanimously, participants favored engagement in their mother tongue. Where this was not possible, most participants were comfortable using a bilingual research assistant or an English speaker with an interpreter. One female said “Since there is a translator and I could understand what he is saying and asking, I don’t think there would be a problem. I would participate”. However, in the Somali group an unexpected finding was that some participants were suspicious of interpreters. Providing a financial incentive was reported as being a motivating factor but many participants were clear to indicate that money alone would not have been sufficient if they were not willing to participate for other reasons.

Unclear communication about the research study may cause participants to perceive involvement in research studies is mandatory. This sense of obligation was associated with increased likelihood of participation. An Arabic female said “I was confused and exhausted after a long trip. The recruitment was like an order to obey. I couldn’t say no”. Alternatively, two aspects of appropriate communication involved in the informed consent process, disclosure and ensuring understanding, were brought up by participants and were associated with increased likelihood of participation. A Somali male summarized this well; “The most important thing to make the participant comfortable, is to do what you did. To explain to us everything in a good manner and make us feel at ease. If he has any questions or private concerns, they should be answered and addressed. So once he is fully informed and convinced that this is not something shady, then he will participate”.

#### 3.1.2. Research Team

Demographic and personal features of the researchers, including their background, gender, familiarity, associations and personal qualities, influenced willingness to participate. Most participants expressed a desire for a researcher from their home country. One Somali female shared a particular concern about local researchers: “When I first came, I had young children with me. So any Caucasian who I saw, I was afraid they would take away my children. That’s what I used to hear from other people”. However suspicion of a researcher with a shared nationality may also exist if refugees are from a region where politics or conflict may have made them suspicious of fellow citizens.

Some participants felt that the gender of the research may influence participation; one Farsi/Dari speaking woman stated “one is more comfortable with women” and a Somali woman noted “some men are shy around women”. Others felt gender was irrelevant. Comments suggested that the importance of gender may be mediated by the nature of the research questions, with more participants preferring the same gender for more sensitive topics.

Personally knowing the researcher was reported as factor that would influence disclosure of personal information and willingness to participate. Other qualities of the researcher, such as their affiliations (particularly with a hospital or settlement center), occupation (particularly being a doctor) or personal qualities were reported as being influential. As a Farsi/Dari speaking woman said “it is in my nature that if I like the person then I would allow it”.

#### 3.1.3. Research Study

Both logistical aspects and the anticipated impact of a study affected willingness to participate. Logistically, with respect to location, several participants indicated that a familiar and convenient place may make them more likely to participate. In the Arabic group there was also a discussion of the impact of hosting research activities in a religious setting such as a mosque. Participants did not feel that a religious setting would alter their decision to participate. Other logistical factor included childcare and time constraints. When discussing the logistics of different types of health research, safety concerns were raised. As would be expected, individuals were less willing to participate in research that might put their health at risk. When one Farsi/Dari speaking female reflected on this, saying “For example, the government now has a new medication, and no one has tried this medication, they ask you, do you want to take this medication? Because it helps human health. Yes or no? Will you accept or not”? Another participant answered, inferring that she would decline, with “well, we are not mice”.

With respect to the broader implications of a study, individuals were motivated to participate if they expected a personal benefit from participating. An opportunity to gain knowledge was a strong motivator for participation. An Arabic female said “maybe it is the curiosity. As I am new in this country I want to know everything”. Participants were more willing to participate in research with topics that were interesting and personally relevant and in projects that had the potential for meaningful outcomes that would contribute to positive change. Altruistic motivations were clearly influential with strong statements of support for research that might benefit other people, particularly other refugees. A Somali female stated she would participate “so we can help our fellow brothers and sisters who will come and are new to this country”.

### 3.2. Individual Factors

#### 3.2.1. Participant Demographics

Most participants indicated that those with a low education level would be less likely to participate in research. As a Farsi/Dari speaking female said “I’m illiterate. I can’t answer questions. I don’t understand. I understand nothing. What am I gonna say when I go there”*?* However, some group members differed and felt that regardless of education level any refugee could participate. A Somali woman said “sure, because you still have a brain, and if you put your mind to it you can change something”.

Previous exposure to war was mentioned by just a few individuals, but in all cases it was felt to decrease willingness to participate in research. As one woman shared “even if there is a translator, if I’m … illiterate and just arrived from Afghanistan, have no language skills and have lived through war conditions where there were guns and rockets, and there were times we couldn’t get a dried piece of bread to eat, my head doesn’t work, I have no business there. I won’t participate”.

Lack of proficiency in the local language was unanimously felt to be a barrier. An Arabic male said “some might feel shy because he afraid of making mistakes in language or in behavior”. An interpreter was a facilitating factor for some but not all participants.

In general, refugees in our study did not see religion as being a factor influencing participation. While a Farsi/Dari speaking woman said “it has to do with the research project. It has nothing to do with being a Muslim or Christian” in one instance a Somali participant did comment that her religion might be a barrier noting “if some Somali girls see her with Caucasian people, they might assume that she has abandoned the religion, and that she wants to assimilate to what they are”.

The impact of a participant’s gender on likelihood of participation was mixed. In the Somali group, there was an uncontested impression that women may be less likely to participate due to shyness and shame. In the Karen group, gender was felt to be irrelevant. In the Arabic group too, there was a passing mention that gender would not be influential and the discussion moved on. In the Farsi/Dari group some too thought that gender was irrelevant, while a group of female participants thought that women were more likely to participate because the issues were more important to them. A male participant agreed, but felt that the reason that fewer men participated was due to their occupational roles and their financial obligations.

#### 3.2.2. Attitudes towards Research

**Figure 1:**
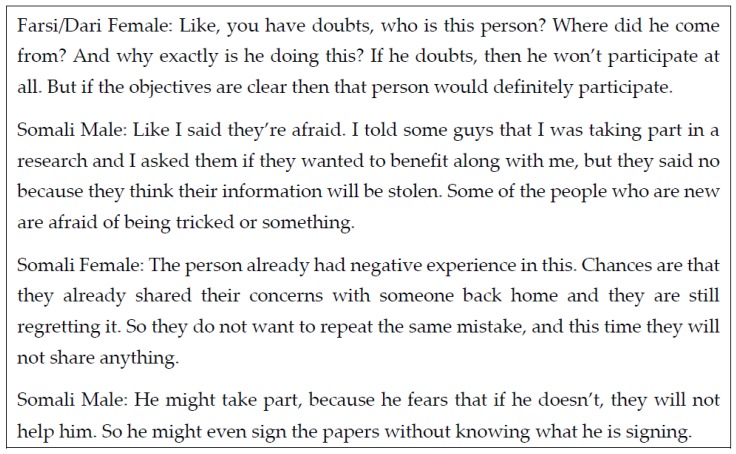
Quotes illustrating fear and suspicion about research.

Participants generally had very positive attitudes towards research which was also associated with an increased likelihood of participation. One Karen male spoke for his group saying “on behalf of us we are very happy to participate in this research study and thank you for doing a research and we would like you to do more research study in the future”. As for negative attitudes, these included fear and suspicion generally about research, fear of loss of confidentiality and fear of consequences of not participating. The level of suspicion and fear was most expressed in the Farsi/Dari speaking and Somali groups, with almost no mention of these concerns in the Karen and Arabic groups. See Figure 1 for some illustrative quotes.

#### 3.2.3. Knowledge about Research and Previous Experience with Research

Participant comments directly and indirectly revealed a diversity in individual participant’s knowledge about research and previous experience with research, but there did not appear to be a consistent link with willingness to participate in research.

## 4. Discussion

Our study of refugees’ perspectives on factors influencing refugees’ willingness to engage in research identified twenty-three variables important to consider in health research. There are no other studies that we are aware of with refugees’ perspectives on research engagement with which to compare our results. Of note, our research findings about refugees are in line with previous findings about the perspectives of ethnic minorities more generally [14,15], however, this study also elicited unique variables not previously identified in the literature.

Notably, this study unearthed three important factors that enhanced the efficacy of recruitment but with ethical concerns. Firstly, if refugees are being recruited into a research study shortly after arrival in their host country, this could result in either lower participation rates due to confusion, fear, and feeling overwhelmed or higher participation rates due to misconceptions about participation being obligatory. In both circumstances, there are ethical concerns due to the risk of exclusion on one hand and of coercion, lack of comprehension, and involuntary participation on the other. As a result, we recommend that researchers try to avoid research recruitment shortly after arrival.

Secondly, if refugees perceive that participation in research is mandatory they are more likely to participate. The perception of mandatory participation seemed most likely to occur if research activities appeared to be related to government activities. Consequently, researchers need to pay particular attention to the power dynamics and the perceptions that may occur as a result of any stated or assumed research affiliations. The voluntary nature of participation must be clearly stated and understood.

Thirdly, participants indicated that a refugee’s fear of consequences of not participating also may inappropriately increase a participant’s willingness to participate at any time. Researchers should be aware of this possibility and make special efforts to inform participants that their health and opportunities will not be impacted by refusal to participate.

There are several recommendations based on the findings of this research. To improve recruitment ethically, researchers should consider logistical factors such as selecting convenient times and locations, providing childcare, and consider providing reasonable financial reimbursement for participants’ time. With respect to communication factors, we recommend ensuring all research material and in-person communication is available in the participants’ own language, providing clear information about the purpose of the research, and ensuring comprehension.

We also recommend engaging research assistants who speak participants’ language and who are good communicators with strong interpersonal skills. For sensitive research topics consider using research assistants who are the same gender as the target population. Engaging assistants who are personally acquainted with potential participants or who are affiliated with health or settlement services may also enhance recruitment, however, one must be cautious that this does not result in coercion leading to involuntary participation.

To maximize the benefit of research participation for refugees, if possible, researchers should consider providing opportunities for participants to learn about health or settlement issues during their participation in the research project.

Based on the findings of our research, researchers should also be aware of the characteristics of refugee subgroups that may correspond to recruitment challenges and corresponding strategies that may aid in engaging these groups. Refugees who have lower education or who are illiterate may have more trouble participating in research. This can be addressed by using appropriate terminology and providing assistance for reading written materials. Refugees who have been exposed to war may also be reluctant to engage in research. Researchers should consider the ethical appropriateness of recruiting this population, and consider the benefits and harms of participation for these individuals. Refugees who for various reasons may be suspicious of research and fear of loss of confidentiality are less likely to engage in research. To address this, researchers can ensure transparency of research procedures, adequate disclosure, opportunities for questions, and diligent attention to maintaining confidentiality. With respect to gender, some female refugee subgroups may be shy or have fear of shame. This may be addressed by utilizing female research assistants. As for males, they may have more concerns about financial and occupation commitments that could be affected by participating in research. This can be addressed by planning appropriate times for research activities and providing financial reimbursements. Of note, the ethical challenges of engaging refugees in research were also addressed directly in this study. Due to the expansive nature of the topic, the methods and results for this aspect of the study are not included within this publication but can be found in depth elsewhere [18].

There were limitations to this study. There was of course a selection bias. Given that the purpose of this study was to understand refugees’ willingness to participate in research, and the fact that sampling was non-random and participation was voluntary, we must recognize that the study sample was in fact a subset of refugees who had already demonstrated their willingness to participate in research. However, we believe that our sample was representative as overall two-thirds of all individuals contacted for this study agreed to participate. Further, comparing the demographics of our study sample to population data shows that our sample was well suited to represent the population of GARs that settled in BC between 2005 and 2009, however our sample had a greater proportion of women.

It is unclear if results of our study can be extrapolated to other refugee populations as only four language groups were included in the study. However, given the similarity of themes between these four diverse populations we suspect that many of our findings are important to consider regardless of source country. Similarly, our study focused on one subset of refugees, government-assisted refugees. While results may be reflective of other refugee subgroups such as refugee claimants, extrapolation of findings should be done with careful reflection of unique migration concerns for each subgroup.

## 5. Conclusions

In conclusion, this is the first study to directly address refugees’ perspectives on willingness to be involved in health research. This study identified twenty-three variables that impact refugees’ willingness to participate in research. Results from this study allowed us to make recommendations about enhancing refugees’ engagement in research in an ethical manner for refugees generally and for subgroups of refugees that may have more difficulties engaging in research. These results and recommendations should be considered for recruitment of refugees in future health research.

## Figures and Tables

**Table 1:** Factors influencing willingness to participate in research.

Research Design Factors		Individual Factors	
Recruitment factors		Demographic variables	
Financial Incentives	Financial incentives increase willingness to participate.	Education level	Low education level may decrease willingness to participate for some, but not all, participants.
Timing	Shorter duration of time in Canada decreases willingness to participate or increases but only due to fear and sense of necessity.	Exposure to war	Exposure to war decreases willingness to participate.
Language	Language concordant research sessions, documents, and research staff or the presence of an interpreter increases willingness to participate.	Lack of local language	Lack of comfort with the local language decreases willingness to participate.
Informed consent	Improved disclosure and enhanced comprehension increase willingness to participate.	Religious beliefs	Religious beliefs generally do not impact willingness to participate in research.
Perception of mandatory participation	If patient perceives research to be mandatory they are more likely to participate.	Gender of participant	The gender of the participant has variable impact on willingness to participate, from no impact, to females being more likely to participate to females being less likely to participate.
Research team factors		Participant attitudes	
Gender of the researcher	The gender of the researcher generally does not matter but some participants prefer same gender researchers, particularly for sensitive topics.	Positive attitudes towards research	Positive attitudes towards research increase willingness to participate.
Nationality of the researcher	The nationality of the researcher generally does not matter but may for refugees with certain political contexts.	Negative attitudes towards research	Fear and suspicion decreases willingness to participate.
Personally knowing the researcher	Personally knowing the researcher increases willingness to participate.		Fear of loss of confidentiality decreases willingness to participate.
Personal qualities of the researcher	Good personal qualities of the researcher, such as friendliness, increase willingness to participate.		Fear of consequences of refusing to participate might increase willingness to participate.
Affiliations or occupation of the researcher	Researcher affiliations with health or settlement organizations, or researchers with trustworthy occupations, increase willingness to participate.		
Research study factors		Participant knowledge and experience with research	
Expected outcomes of the study	Opportunities that might benefit participants or others; acquiring knowledge; and believing that the purpose of the study is meaningful increases willingness to participate.	Previous knowledge and experience with research	Participant knowledge and experience with research did not impact willingness to participate in research.
Logistical factors	A practical location and the availability of childcare increase willingness to participate.		
	Time constraints can decrease willingness to participate.		
	Beyond practicality, the location of the research does not seem to matter.		
Safety concerns	Any research that puts participants at risk decreases the likelihood of participation.		
